# Uncovering the role of ABI2 as a key regulator of flowering time in Arabidopsis

**DOI:** 10.1080/15592324.2026.2684387

**Published:** 2026-06-06

**Authors:** Akhtar Ali, Shah Zareen, Junghoon Park, Dae-Jin Yun

**Affiliations:** a School of Advanced Biotechnology, Global Plant Stress Research Center, Konkuk University, Seoul, South Korea

**Keywords:** Flowering time, ABA signaling, ABI2, SnRK2s, ABI5, FLC

## Abstract

Abscisic acid (ABA) is a fundamental regulator of plant development, growth, and drought adaptation, and it modulates the transition to flowering. However, the molecular mechanisms by which ABA delays flowering remain unclear. Recently, ABA INSENSITIVE 2 (ABI2), a core component of the ABA signaling pathway, was reported to positively regulate floral transition in *Arabidopsis*. The expression of floral repressors such as *FLC* and *CDF1* was strongly upregulated, whereas flowering promoting genes including *FT*, *SOC1* and *CO* were downregulated in the *abi2-2* mutant. Moreover, ABI5 protein levels and phosphorylation status were enhanced in *abi2-2*, suggesting that ABI2 reduces ABI5 activity and suppresses the activation of its target genes, such as *FLC*. Genetic analyses revealed that ABI2 functions upstream of ABI5. In conclusion, these findings indicate that ABI2 is a major switch that fine-tunes the crosstalk between ABA signaling and floral transition in *Arabidopsis*.

## Introduction

Adverse conditions, such as high salinity, cold, and drought stress, are critical challenges in agriculture because they reduce the crop yield.^1^ Phytohormones play a vital role in stress management by inducing various biochemical and physiological changes.^2-7^ Although the abscisic acid (ABA)-mediated regulation of seed maturation, embryo morphogenesis, stomatal movement, and floral transition has been extensively studied,^3^ ^,^ ^8-10^ the molecular mechanism by which ABA delays flowering remains unclear. In recent years, the relationship between ABA signaling and flowering pathways has emerged a significant area of research focus. *FLOWERING LOCUS C* (*FLC*)-mediated temperature-dependent seed germination occurs through downstream flowering pathway genes, including the *FLOWERING LOCUS T* (*FT*), *SUPPRESSOR OF OVEREXPRESSION OF CO 1 (SOC1)*, and *APETALA 1 (AP1)*, establishing FLC as a major regulator of this process.^11^ In addition, *ABA-Responsive Element-Binding Factors* (*ABFs*), a subgroup of bZIP transcription factors, participate in ABA signal transduction during seed germination and in vegetative stress responses.^12^ ^,^ ^13^ *ABF1, ABF3*, and *ABF4* are predominantly expressed in vegetative tissues, whereas *ABA-INSENSITIVE 5 (ABI5)*, a subgroup A bZIP transcription factor, is preferentially expressed during seed maturation and germination.^14-16^

Seed germination and flowering are transitional phases that respond to similar seasonal signals. However, the detailed mechanisms by which these two complex pathways modulate each other remain unknown. Recently, *ABI5*, which plays an important role in ABA-mediated seed arrest,^17^ was found to be a major regulator of *FLC* expression.^18^ ABI5 directly binds to the FLC promoter and activates its transcription, resulting in delayed flowering.^18^ In addition, *ABI4* negatively regulates flowering by activating *FLC* transcription,^19^ highlighting the importance of ABI4 and ABI5 in the ABA-mediated delay of floral transition. OPEN STOMATA1 (OST1), an SNF1-related kinase, suppresses flowering by phosphorylating and activating ABI5, which is crucial for the ABA-dependent transcriptional activation of *FLC*.^18^ Based on current evidence, *ABA-INSENSITIVE 4 (ABI4)* and *ABI5* are the only ABA-responsive transcription factors involved in the regulation of floral initiation within the canonical ABA signaling pathway. However, whether additional core ABA components directly modulate flowering time remains unknown. In this context, the roles of ABA-signaling clade A type 2 C protein phosphatases (PP2Cs), such as ABA INSENSITIVE 2 (ABI1) and ABI2, which inhibit SnRK2s activity and their downstream transcription factors, are particularly relevant.^20-22^ Exploring the mechanisms by which PP2Cs modulate SnRK2-ABI5/ABI4 function is crucial for elucidating the role of ABA in the flowering regulation network. Recent findings indicate that ABI2 promotes flowering by negatively regulating the OST1-ABI5-FLC module under long day/short day (LD/SD) conditions.^23^

## ABI2 promotes floral transition

**Figure 1.**
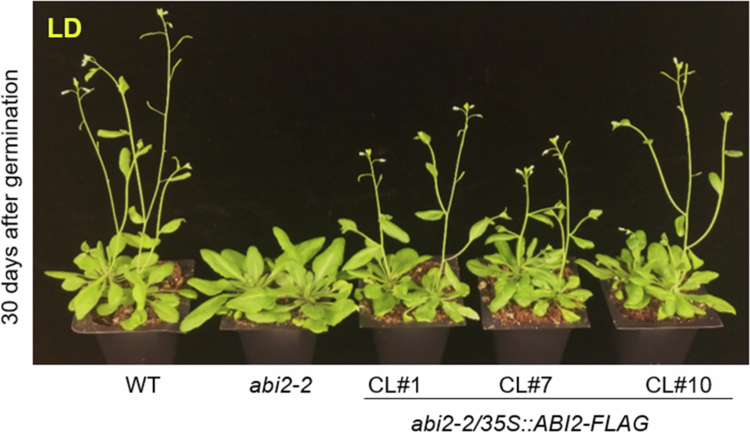
*abi2-2* mutant exhibits a late-flowering phenotype. The loss-of-function *abi2-2* mutant exhibited late flowering, which was rescued by *ABI2* overexpression (*35S:ABI2-FLAG*). Plants were grown under long-day growth conditions (LD).

ABA regulates flowering by either accelerating or delaying the floral transition, depending on the developmental and environmental contexts.^24^ As a negative regulator, ABA delays flowering under unfavorable conditions by activating ABI5, which induces *FLC* expression and represses flowering under LD conditions.^18^ In addition to *ABI5*, *ABI4* promotes *FLC* transcription, and *ABA INSENSITIVE 3 (ABI3)* acts through *CONSTANTS (CO)*, thereby contributing to ABA-mediated repression of flowering.^19^ ^,^ ^25^ In contrast, ABA can act as a positive regulator by accelerating the expression of florigen genes, such as *FT*, *TWIN SISTER OF FT (TSF)*, and *SOC1*, to promote flowering in response to drought.^24^ For instance, ABF3 and ABF4 cooperate with NF-Y complexes to activate the expression of *SOC1*.^26^ Similarly, ABI2 has been identified as a unique core ABA-signaling phosphatase that promotes flowering under both LD and SD conditions.^23^ *abi2-2* mutant lines exhibit a late-flowering phenotype, as evidenced by an increased number of rosette leaves and delayed bolting and flowering.^23^ In addition, FT expression was downregulated in *abi2-2* lines compared to that in wild-type (WT) plants, supporting the delayed flowering phenotype of *abi2-2*.^23^ To further confirm the functional role of ABI2 in flowering transition and assess whether it restored the flowering phenotype of *abi2-2*, we introduced the full-length *35S:ABI2-FLAG* construct into *abi2-2* mutant background plants. The introduction of *35S:ABI2-FLAG/abi2-2* fully rescued the late-flowering phenotype of the *abi2-2* mutant (Figure 1), confirming the role of AB12 in promoting floral transition. *ABI2* transcript levels show only modest circadian variation under LD conditions, with a slight increase in the morning, suggesting that ABI2 is constitutively available to modulate flowering time.^23^ These findings indicate that ABI2 is a major positive regulator of floral transition in *Arabidopsis*, acting within the ABA signaling network to promote timely flower development.

ABI2 and ABI1 represent highly homologous phosphatases that exert negative regulation over SnRK2 kinases and their target substrates via dephosphorylation;^27^ however, only ABI2 has been identified as a regulator of flowering time.^23^ Another report stated that the inhibition of BR signaling by ABA relies primarily on ABI2, with a partial contribution from ABI1.^28^ These findings suggest that ABI1 and ABI2 may possess distinct substrate preferences and interaction dynamics, as well as tissue- or developmental stage-specific expression patterns and differential regulation by ABA receptors or interacting partners within floral tissue. Nevertheless, the precise molecular mechanisms underlying the unique role of ABI2 in flowering, as compared to ABI1, have not yet been fully elucidated. Future research comparing the interaction networks, substrate specificities, and regulatory mechanisms of ABI1 and ABI2 in flowering tissue will be essential to resolve this matter.

## ABI2 exerts differential regulation over the expression of flowering pathway genes

In recent years, several ABA signaling components have been shown to regulate flowering time, either positively or negatively.^24^ The ABA-dependent regulation of the circadian clock gene *GIGANTEA (GI)* facilitates drought escape (DE) mechanisms by modulating *FT*.^29^ ^,^ ^30^ The OST1 homolog SlOST1 in tomato, accelerates flowering by phosphorylating the NAC-type transcription factor vascular plant one-zinc finger 1, which binds to the *SINGLE FLOWER TRUSS* (*SFT*) promoter to activate its expression.^31^ In *Arabidopsis*, the *snrk2.2/2.3/2.6* triple mutant flowers earlier than WT plants, accompanied by increased expression of *LEAFY (LFY)*, *FLOWERING LOCUS D (FD)*, and *AP1*, and reduced *FLC* levels.^18^ ABA-dependent transcription factors, including *ABF3*, *ABF4*, *ABI5*, *ABI3*, and *ABI4*, modulate floral transition by altering the expression of florigens, sssuch as *FT*, *TSF*, and *SOC1*, or by promoting *FLC* expression to delay flowering.^18^ ^,^ ^19^ ^,^ ^25^ ^,^ ^26^ ABI2 serves as a fundamental element within the ABA signaling pathway, it also functions as a positive regulator of flowering,^32-34^ which correlates with the altered expression of flowering time genes.^23^ FLC, a key repressor of flowering, is activated by ABA-dependent transcription factors, such as *ABI5*, *ABI3*, and *ABI4*, with *ABI5* and *ABI3* forming a complex at the *FLC* promoter to induce its expression.^24^ To dissect how ABI2 controls the transcript levels of key flowering regulators, expression levels were compared between the WT and *abi2-2* mutant plants. The expression of *FLC* and *CYCLING DOF FACTOR 1 (CDF1)*, repressors of *CO* and *FT*, was strongly upregulated in *abi2-2* compared with that in WT plants,^23^ indicating that ABI2 suppresses *FLC* and *CDF1* expressions. Consistent with this, the expression of *CO, FT*, and *SOC1* was significantly downregulated in *abi2-2* plants compared to that in WT plants.^23^ Overall, these transcriptional changes align with the late-flowering phenotype of *abi2-2* and support the conclusion that ABI2 is a key positive regulator of the flowering pathway that promotes the flowering transition primarily by suppressing FLC and modulating its downstream networks.

## ABI2 functions as a molecular switch of ABA signaling to flowering time

**Figure 2.**
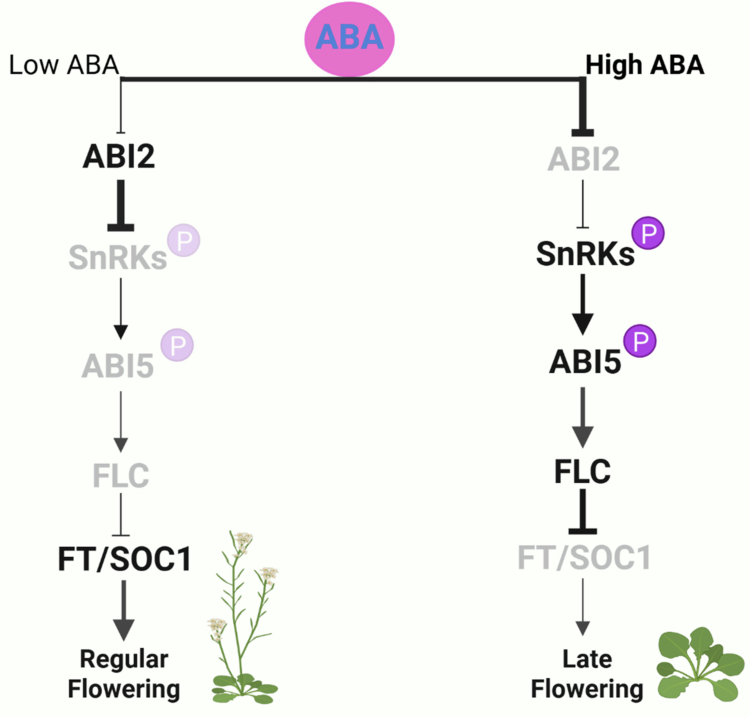
Proposed working model. Under standard growth conditions, characterized by low ABA levels, ABI2 inhibits the activity of SnRK2s via dephosphorylation. This process subsequently leads to a reduction in ABI5 activation and diminished regulation of FLC, which reverses the downregulation of *FT* and *SOC1*, promoting regular flowering. Under high ABA conditions, ABI2 is inhibited, leading to the activation of SnRK2s via autophosphorylation. Activated SnRK2s then phosphorylate and activate ABI5, thereby enhancing FLC expression. FLC subsequently represses FT and SOC1, resulting in late flowering.

Previous studies have shown that ABI5 directly binds to the *FLC* promoter to activate its transcription, thereby delaying the floral transition under LD conditions.^18^ Although ABI5 mRNA expression increased modestly after ABA treatment, its protein level increased significantly, indicating that ABI2 primarily influences ABI5 at the posttranslational level.^23^ In contrast, ABI5 protein levels and phosphorylation were downregulated in *ost1-3* plants,^23^ suggesting that OST1 promotes ABI5 stability, possibly through phosphorylation. ABI5 phosphorylation was induced in *abi2-2* plants following ABA treatment,^23^ suggesting that ABI2 suppresses OST1-mediated ABI5 phosphorylation and stabilization, thereby indirectly inhibiting *FLC* expression (Figure 2). Consistent with this, *FLC* expression is downregulated in *abi5* and *snrk2* mutants, and ABI5 requires OST1-dependent phosphorylation to activate *FLC* transcription.^18^ Given that *FLC* mRNA expression is strongly induced in *abi2-2* plants,^23^ we proposed that the late-flowering phenotype of *abi2-2* is due to the OST1- and ABI5-dependent hyperactivation of FLC. Moreover, the late-flowering phenotype of *abi2-2* was completely rescued in *abi2/ost1* and *abi2/abi5* double-mutant plants, and the expression of *FLC* was downregulated in these double mutant plants.^23^ In addition, the late-flowering phenotype of *abi2-2* was completely rescued in the *abi2-2/flc-3* double mutant, ^23^ confirming that FLC is the main downstream effector of ABI2-mediated regulation of flowering. These genetic and molecular data indicate that ABI2 positively regulates flowering by inhibiting OST1- and ABI5-mediated *FLC* transcription and that ABI2 acts as an upstream molecular switch in the common pathway with OST1, ABI5, and FLC to fine-tune flowering initiation in response to ABA.

## Summary and future perspective

Despite previous evidence that ABI5 and ABI4 directly activate FLC transcription to delay flowering,^18^ ^,^ ^19^ the ABA-dependent regulatory plasticity of the floral transition under stress requires careful consideration. Elevated ABA levels can enhance this mechanism by activating bZIP transcription factors, such as ABI5, whose activation requires ABA-activated SnRK2 kinases under stress conditions (Figure 2).^18^ ^,^ ^19^ OST1-mediated activation of ABI5 promotes FLC expression, thereby suppressing flowering (Figure 2).^23^ To reverse the normal developmental timing after stress, plants likely require rapid attenuation of the ABI5-FLC module via ABI2-dependent dephosphorylation and the destabilization of ABI5 (Figure 2). Consistent with this notion, the phenotypes of the double mutants *abi2/ost1*, *abi2/abi5*, and *abi2/flc* indicate that ABI2 functions as a major positive regulator of flowering by indirectly suppressing FLC via the inhibition of OST1 and ABI5.^23^ However, the mechanisms by which the single PP2C phosphatase ABI2 interacts with other partners and flowering time regulators to fine-tune floral transition remain unclear. Future studies should examine whether ABI2-dependent regulation of flowering initiation is conserved in crop species and explore this regulatory module to optimize reproductive timing under fluctuating environmental conditions.
